# Dorsal column mapping in resection of intramedullary spinal cord tumors: a prospective comparison of two methods and neurological follow-up

**DOI:** 10.1007/s00701-023-05554-1

**Published:** 2023-03-17

**Authors:** Moritz Ueberschaer, Katharina Breitkopf, Sebastian Siller, Sophie Katzendobler, Jonathan Weller, Tobias Greve, Stefan Zausinger, Joerg-Christian Tonn, Andrea Szelenyi

**Affiliations:** 1 Department of Neurosurgery, Ludwig-Maximilians-University, Marchioninistr. 15, 81377 Munich, Germany; 2 Isar-Amper-Klinikum München-Ost, Haar, Germany

**Keywords:** Intramedullary spinal cord tumors, Intraoperative neuromonitoring, Dorsal column mapping, Midline myelotomy

## Abstract

**Purpose:**

In surgery for intramedullary spinal cord tumors (imSCT), distortion of the anatomy challenges the visual identification of dorsal columns (DC) for midline myelotomy. Dorsal column mapping (DCM) and spinal cord stimulation (SCS) can identify DC neurophysiologically. We compare application and feasibility of both methods.

**Methods:**

Patients with surgically treated imSCT were prospectively included between 04/2017 and 06/2019. The anatomical midline (AM) was marked. SSEPs at the DC after stimulation of tibial/median nerve with an 8-channel DCM electrode and cortical SSEP phase reversal at C3/C4 after SCS using a bipolar concentric probe were recorded. Procedural and technical aspects were compared. Standardized neurological examinations were performed preoperatively, 1 week postoperatively and after more than 12 months.

**Results:**

The DCM electrode detected the midline in 9/13 patients with handling limitations in the remaining patients. SCS was applicable in all patients with determination of the midline in 9/13. If both recordings could be acquired (6/13), concordance was 100%. If baseline SSEPs were poor, both methods were limited. SCS was less time-consuming (*p* = 0.001), cheaper, and easier to handle. In 92% of cases, the AM and neurophysiologic midlines were concordant. After myelotomy, 3 patients experienced > 50% reduction in amplitude of SSEPs. Despite early postoperative worsening of DC function, long-term follow-up showed significant recovery and improvement in quality of life.

**Conclusion:**

DCM and SCS may help confirm and correct the AM for myelotomy in imSCT, leading to a favorable long-term neurological outcome in this cohort. SCS evolved to be superior concerning applicability, cost-effectiveness, and time expenditure.

**Supplementary Information:**

The online version contains supplementary material available at 10.1007/s00701-023-05554-1.

## Introduction

### Background

Intramedullary spinal cord (imSCT) tumors are rare accounting for 5–10% of all spinal tumors. Cervical and thoracic astrocytomas and ependymomas are most frequent, with young males predominating [8, 19].

Patients usually suffer from local pain or neurological deficits. At this point, most tumors have already grown to a significant size leading to a distortion of the regular anatomy of the spinal cord and its surface. Surgery is the first treatment for imSCT [4, 9–11, 13, 22].

A midline myelotomy is considered as the safest and thus most frequent surgical approach for imSCT [7, 25]. The dorsal median sulcus represents the midline being located between the right- and left-sided dorsal columns (DC) which consist of the medial lemniscus pathway (gracile and cuneate tract) conducting sensory afferents of fine touch, vibration, two-point discrimination, and proprioception (position). Additionally, the middle between the root entry zones and the dorsal median sulcal vein can indicate the midline. Edema, spinal cord rotation, or sheer volume effects may hamper the visual identification of the midline for myelotomy in imSCT. This can lead to surgical DC injury and might contribute to a relevant percentage of the reported rate of 43–55% postoperative ataxia [2, 13, 20].

Intraoperative neuromonitoring (IONM) has been shown to reduce the surgical risk for neurological impairment in imSCT [12]. Thus, standardized IONM with continuous monitoring of somatosensory evoked potentials (SSEP) and motor evoked potentials (MEP) is deemed mandatory [5, 24]. Electrophysiological determination of the DCs may help the surgeon to identify the dorsal midline of the spinal cord for myelotomy.

Two methods for dorsal column mapping (DCM) have been described to reduce the occurrence of postoperative ataxia [15].

1. Recording of spinal somatosensory evoked potentials (spinal SSEP)
  This method was first described by Deletis and Bueno De Camargo [3], and the term “dorsal column mapping” was coined. During tibial nerve stimulation, spinal SSEPs are recorded with an 8-channel electrode (for details, see “Material and methods”) which is placed on the dorsal spinal cord and records conducted SSEPs with the amplitude gradient indicating the topographic anatomy [3, 17, 18].
2. Direct dorsal column stimulation
  Direct stimulation of the posterior spinal cord activates the dorsal column among other pathways. This stimulation evokes somatosensory potentials recorded at the scalp (C3′, C4′, Cz′, and Fz), which show a phase reversal when moving the stimulation from the left dorsal columns to the right side or vice versa. The point where there is no response, between the two opposite phases, indicates the midline [16, 23].

### Objective

The aim of this study was to compare both methods in terms of their feasibility and reliability for correct determination of the anatomical midline, as well as in terms of clinical outcome and cost-effectiveness. Moreover, we correlated the surgeon’s intraoperative anatomical localization of the median raphe with the electrophysiological information.

## Material and methods

### Study design

In this prospective single center study, all patients with imSCT undergoing tumor resection via midline myelotomy were included between April 2017 and October 2019. Patients or patient caregivers had to give informed written consent. The study has been approved by the local ethics committee (735–16).

### Clinical examination

Clinical examination was performed preoperatively, on day seven after surgery and at least after 12 months. The following established and validated questionnaires and examination protocols were used with focus on neurological function of the DC and quality of life (QoL):

- SARA (scale for the assessment and rating of ataxia) score (range 0–40) assesses ataxic symptoms and has been validated for other conditions in addition to its initial indication for assessing spinocerebellar ataxia [21]. The higher the score, the more severe the deficits.
- JOA (Japanese Orthopaedic Association) score (range 0–17) is a myelopathy score that pays particular attention to the extent and location of epicritical sensory disturbances (35.4% of the total score) [6]. The lower the score, the more severe the deficits (normal function ≥ 16; grade 1: 12–15; grade 2: 8–11; grade 3: ≤ 7).
- McCormick score [14] (McS) is another myelopathy score (range 1–4) that allows evaluation of functionality in everyday life. The higher the score, the more severe the deficits (1 = normal function/mild deficit, independent patient; 2 = moderate deficit, independent patient; 3 = severe deficit, dependent patient; 4 = completely dependent patient).
- The pallesthesia score according to Rydel and Seiffer assesses essential function of the DC with a scoring range 0–8 (8 = normal function; 0 = no function) [1].
- Short form-36 (SF-36) QoL questionnaire [26] is a 36-item questionnaire about the patients’ health status, consisting of 8 sections covering different domains of life.

### Intraoperative neurophysiological monitoring

IONM consisted of MEP and SSEP. For recording and stimulation, a commercially available multichannel neuromonitoring device was used (ISIS; Inomed, Emmendingen, Germany).

#### Standard SSEP

For intraoperative monitoring of SSEP, subcutaneous needle electrodes were placed at the medial malleolus for tibial nerve stimulation. Median nerve stimulation was performed with subcutaneous needle electrodes being placed at the wrist. Both nerves were stimulated with a supramaximal intensity of 20–40 mA, an individual pulse width of 0.5 ms for tibial SSEPs and 0.2 ms for median nerve SSEPs, and stimulation frequency of 3.3–4.3 Hz, respectively. Tibial and median nerve SSEPs were recorded with at least two recording montages (C3′, C4′, and Cz′ each referenced to Fz and C3′-C4′ and vice versa). The recording was obtained with a band-pass filter of 50–2000 Hz, a sweep length of 100 ms, and 200 averages/response. Amplitude decrements of more than 50% and latency increments of > 10% of baseline values were considered as warning criteria and interpreted as significant deterioration if they persisted to the end of the surgery.

### Dorsal column mapping (DCM)

Two methods were used to identify the DCs. Electrophysiological data were documented by a standardized protocol (supplement). Data were saved for subsequent review. Stimulation was always started over the center of the tumor and extended to the cranial and caudal parts of the tumor when no reliable measurement could be generated, as suggested in a previous study [15]. The cost calculation included costs of purchase and sterilization.

#### DCM with recording of spinal somatosensory evoked potentials (spinal SSEPs)

##### Stimulation

The same stimulation parameters were used as described for standard SSEP.

##### Recording

**Fig. 1:**
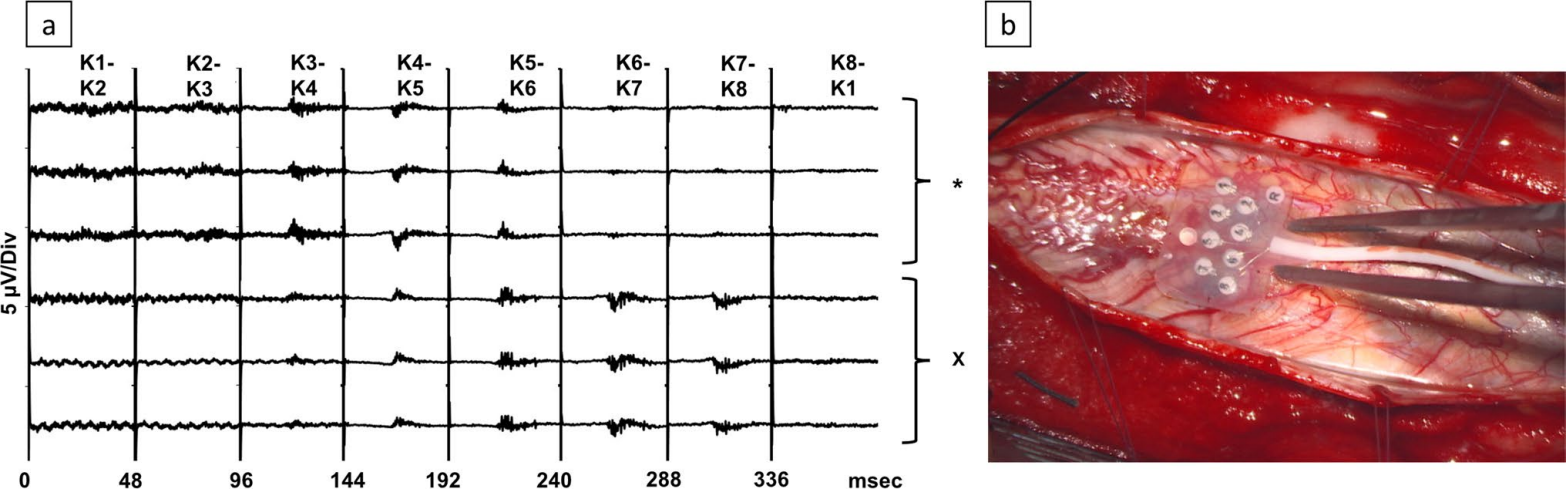
**a** Amplitude gradient indicating the midline after left (*) and right (X) tibial nerve stimulation. Midline localization under electrode 5. **b** Correlating intraoperative image on the right

A multielectrode grid (“M”-Style Contact, AdTech Co.®, USA) was placed on the dorsal surface of the spinal cord. The grid is made up of 8 diagonally offset platinum electrodes of 2 mm diameter with 1.17 mm exposure and 2 mm contact spacing embedded in silastic (Fig. 1). The recording was obtained in a bipolar fashion (1–2, 2–3; 3–4, 4–5, 5–6, 6–7, and 7–8) and a monopolar fashion with Fz serving as reference. The recording was obtained with a band-pass filter of 50–2000 Hz, a sweep length of 100 ms, and a maximum of 200 averages/response. Polyphasic responses were considered as spinal SSEP, whereas a biphasic high amplitude response was considered a dorsal root action potential (DRAP). DRAP can occur when depolarizations are large enough to reach a threshold in the primary afferent endings. This can lead to retrograde propagation (antidromic) of dorsal root reflexes back out the dorsal root and peripheral nerve to the sensory terminals in peripheral tissue [27].

##### Data analysis

For each stimulation site, the amplitude gradient of the spinal SSEP indicated the midline which was determined between the two electrodes showing the highest spinal SSEP amplitude following right and left tibial nerve stimulation. In cervical imSCT, median or ulnar stimulation was used to determine the midline if tibial nerve SSEPs were not present. In thoracic imSCT, the method was considered as “non-recordable” if tibial nerve SSEPs were not present.

#### DCM with direct dorsal column stimulation (SCS)

##### Stimulation

A bipolar concentric electric simulation probe (Inomed®, Germany) was used to stimulate the dorsal surface of the spinal cord in a systematic fashion from right to left. Stimulation was performed with 0.2 s and a maximum of 2 mA and 3.3 Hz repetition rate.

##### Recording

For cortical recording from the scalp, C3′ referenced to C4′ was used and obtained with a band-pass filter of 50–2000 Hz, a sweep length of 100 ms, and a maximum of 100 averages/response.

##### Data analysis

**Fig. 2:**
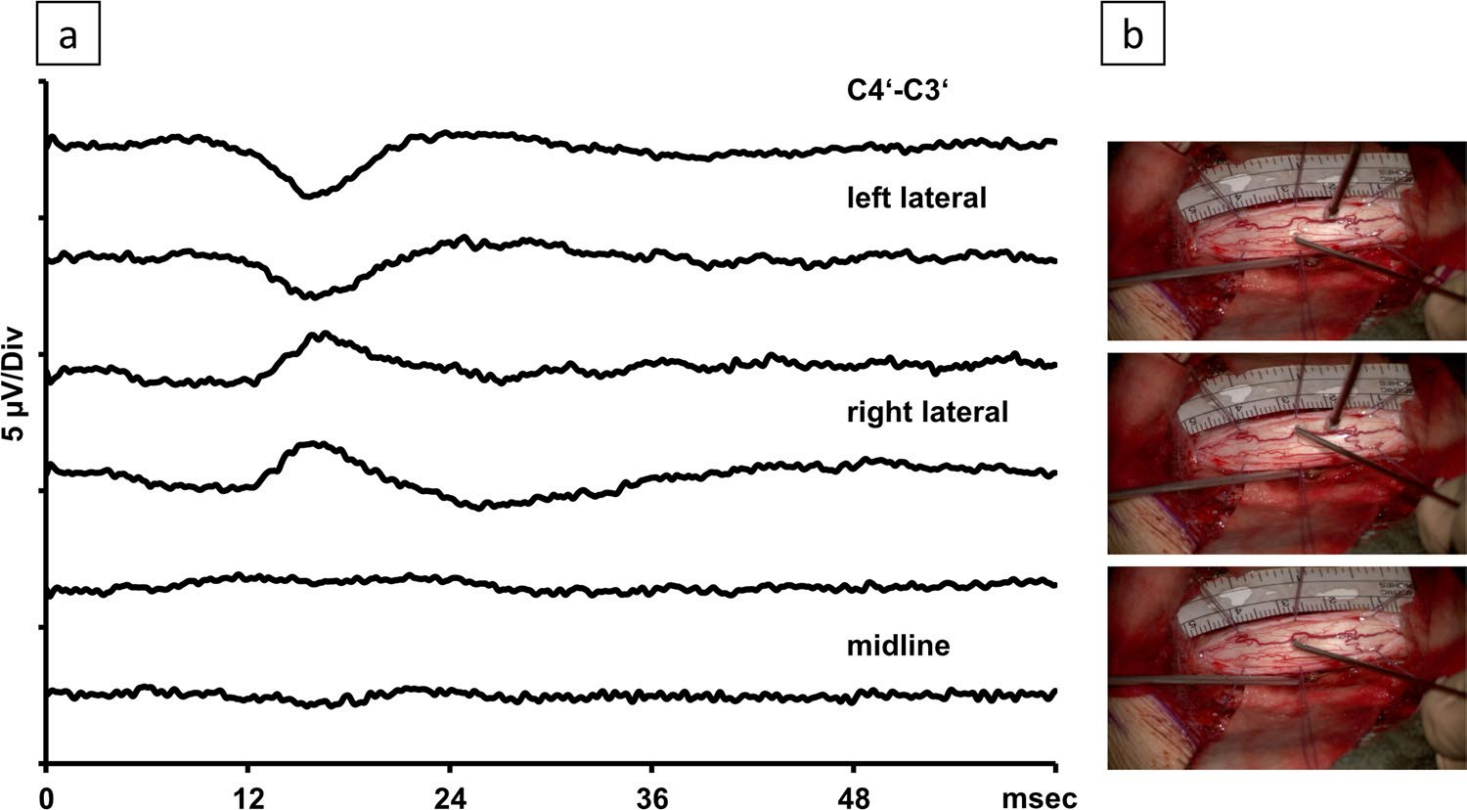
**a** Phase reversal measured at the skull (C4′-C3′) indicating the midline. **b** Correlating intraoperative image on the right

Cortical phase reversal and amplitude changes of SSEPs were used to electrophysiologically identify the laterality of the DCs (Fig. 2). Phase cancelation of the cortical SSEP indicated the electrophysiological midline.

#### Intraoperative study protocol

**Fig. 3:**
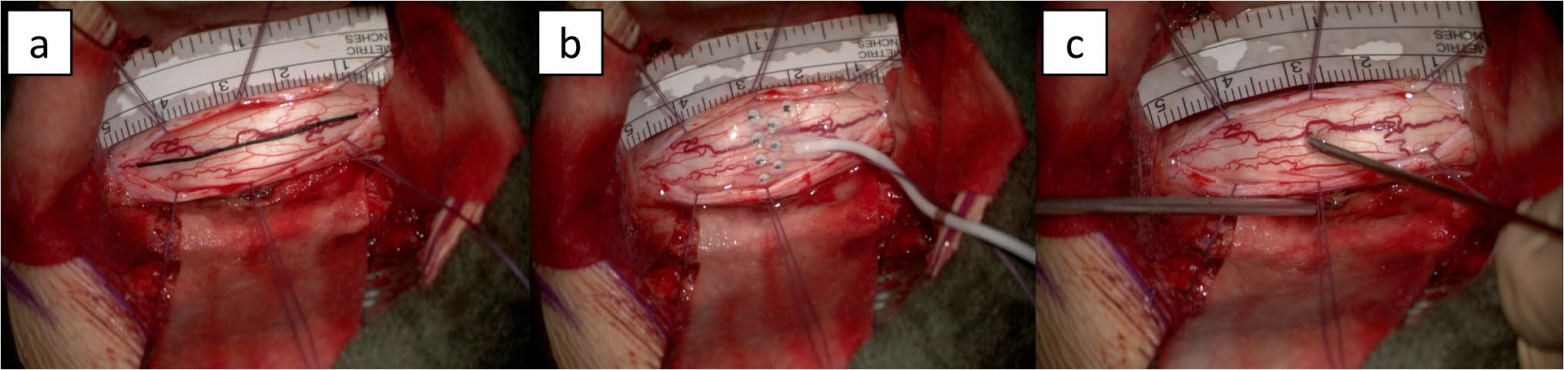
Intraoperative workflow. a Anatomical midline marked by a silk thread. b Grid electrode. c Handheld stimulation device

Anatomical identification of the midline was performed and marked by a silk thread together with a scale. This was photographed and video documented for correlation of the anatomical midline with the electrophysiological midline. Then, the grid electrode was placed and spinal SSEPs were recorded, followed by the direct SCS (Fig. 3). Midline myelotomy by sharp dissection was guided by electrophysiological information after correlation with the intraoperative anatomy. During myelotomy, SSEPs were continuously and concurrently recorded. Any alteration was immediately announced to the surgeon. Changes of amplitudes were announced in percentage of the baseline amplitude and latency changes in millisecond.

After each procedure, the surgeon was asked to indicate his/her preference with regard to handling of the two devices. Any side effects were documented.

#### Statistical analysis

Statistical analysis was performed with SPSS Statistics (24; IBM). A comparison of clinical variables pre- and postoperatively and electrophysiological parameters (maximum peak-to-peak amplitude of spinal and cortical SSEP) was performed using the chi-square test for categorical variables, *t*-test for parametric variables, and Mann–Whitney *U*-test for nonparametric variables. A significance level with *p* values below 0.05 was used to determine statistical significance.

Calculation of the distance between the anatomic and electrophysiological midlines was performed on the intraoperative photographic documentation using the aequo 1.9.1 on-screen measurement tool (https://sebseager.com/aequo). Millimeter resolution is possible by referencing with the intraoperative scale. If the deviation was more than 1.5 mm, the midline localization was rated discordant considering the spatial resolution of both tools.

## Results

### Patients and clinical data

Thirteen patients (8 female, 5 male) with a median age of 43 years (15–79) were included. For detailed patient characteristics, see Table 1.

**Table 1:** Sex, age, histology, localization of the pathology, and preoperative clinical score by individual patient

Sex	Age	Histopathology	Location	McCormick score	SARA score	JOA score
F	23	Ependymoma WHO °II	C 5/6	0	0	17
F	53	Diffuse midline glioma WHO°IV	C 2	3	10.5	3.5
F	34	Ependymoma WHO °II	C 4/5	1	0	15
M	41	Arachnoid cyst	C 1–5	2	14	12.5
M	15	Diffuse midline glioma WHO°IV	C 3–7	2	0	16
F	29	Cavernoma	Th 10	2	2	12.5
F	58	Ependymoma WHO°II	C 5–6	0	0	17
M	62	Ependymoma WHO°II	Th 6	2	8	7
M	65	Ependymoma WHO°II	C 1	1	0	17
F	39	Ependymoma WHO°II	C 3	0	0	17
F	79	Arachnoid cyst	Th 10	2	7	11.5
M	18	Ependymoma WHO°II	Th 2–3	0	1	17
M	45	Ependymoma WHO°II	C 3–7	1	4	13

### Intraoperative neuromonitoring with standard SSEPs

Baseline tibial nerve SSEPs were present in 12/13 (92%) and median nerve SSEPs in 11/13 (85%) patients without any changes during the surgical approach. Accordingly, SSEP monitoring with respect to the spinal level was possible in 12/13 (92%) patients. Detailed information on SSEPs at baseline, after myelotomy, and at the end of surgery is provided in Table 2. Regarding tumor location, baseline tibial SSEPs were present in 8/9 (89%) cervical and 4/4 (100%) thoracic tumors, and baseline median SSEPs were present in 7/9 (78%) cervical and 3/4 (75%) thoracic tumors. Neither transient deteriorations nor transient or permanent losses of SSEPs occurred. A persistent deterioration of amplitudes > 50% was observed in 3/12 (25%) patients related to myelotomy. In two of these patients, the electrophysiological midline was identified by both methods, in one patient only by SCS. In all three cases, the anatomical and electrophysiological midline coincided (Table 3).

**Table 2:**
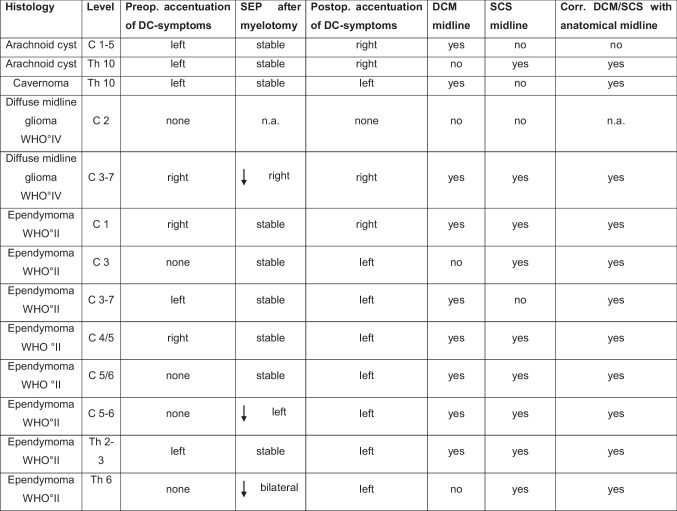
Comparison of clinical and electrophysiological aspects and anatomical correlation of myelotomy after DCM. *DC*, dorsal column; *arrow down* (*↓*), SSEP amplitudes < 50% of baseline SSEP

**Table 3:**
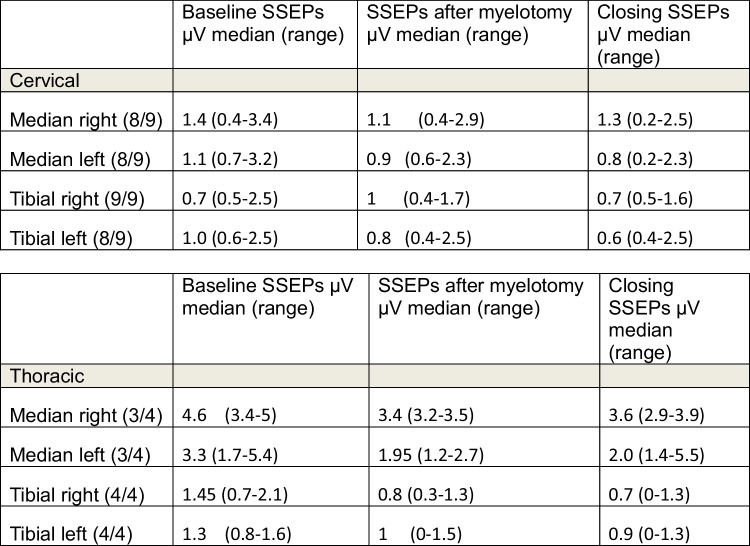
SSEP of upper and lower extremities over the course of the surgery, divided according to the location of the tumors

During tumor resection, in another two patients, persistent reduction of amplitudes > 50% of tibial nerve SSEPs occurred.

### Recording of spinal somatosensory evoked potentials (spinal SSEPs) for midline detection

In 9/13 (69.2%) patients, spinal SSEPs demonstrated an amplitude gradient indicating the midline. Regarding tumor location, midline detection was possible in 7/9 (77.8%) cervical and 2/4 (50%) thoracic tumors. Median time for implementation and measurements was 17 min (range 6–23 min).

In 3/13 (23%) patients, the electrode could not be placed satisfactorily on the spinal cord due to the lack of elasticity of the grid or the connected cable or lack of space. In 1/13 (8%) patients, the measurement did not allow a reliable indication of the midline, since only altered potentials (DRAP) could be derived and tibial SSEPs were absent. The location of the tumor in the upper cervical spine above C3 correlated with non-feasibility of implementation and measurement (*p* = 0.019), while tumor histology or morphology (cystic vs. solid) did not.

### Recording of direct dorsal column stimulation (SCS) for midline detection

In 9/13 (69.2%) patients, the midline could be identified by a typical phase reversal. In all these patients, we also registered a flattening of the amplitudes (phase cancelation) when the dorsal median raphe was stimulated directly. Concerning tumor location, midline detection was possible in 6/9 (66.7%) cervical and 3/4 (75%) thoracic tumors.

Median time for implementation and measurement was 9 min (range 4–13 min).

In 4/13 (31%) patients, SCS failed to identify the midline, as only stimulation of one DC could provide a reliable response. Three of these patients had tumors in the upper cervical spine near the craniocervical junction (one ependymoma, two astrocytomas). One patient had a recurrent cavernoma in the lower thoracic spine. Tumor recurrence correlated with measurement failure (*p* = 0.019), but tumor location and tumor histology and morphology did not.

### Identification of the anatomical midline and comparison with neurophysiological data

The anatomical midline was identified by the surgeon in all 13 (100%) patients using the above-mentioned landmarks and by at least one electrophysiological method in 12/13 (92%) patients. Concordance between the electrophysiological midline with the anatomical midline was high (11/12 cases (92%)).

There was one patient that showed a discordance between the anatomical and electrophysiological midline (2.5 mm) as determined by spinal SSEP. This patient had no tibial and median SSEPs of the left side preoperative, and the direct DC stimulation could not determine the electrophysiological midline.

In this case, the electrophysiological midline was chosen for myelotomy after reevaluation of the anatomy. After myelotomy, there was no worsening of SSEPs in this patient.

### Comparison of spinal SSEP and direct spinal cord stimulation methods

In 12/13 (92%) patients, at least one electrophysiological method allowed determination of the midline. In 6/13 (46%) patients, both methods equally provided reliable identification of the electrophysiological midline with 100% concordance. 2/6 (33%) tumors were located thoracically, 4/6 (67%) cervically. All patients had baseline tibial SSEPs.

In 3/13 patients (23%), only SCS and, in another 3/13 patients (23%), only spinal SSEP could indicate the midline. In one of these six patients, the DCM identified the midline 2.5 mm more lateral of the anatomical midline.

In one patient with a diffuse midline glioma at the level of C2, the midline could not be reliably determined with either method. This patient already suffered from severe neurological impairment preoperatively and had poor SSEPs.

#### Procedural aspects

Implementation of the electrodes and measurement were significantly shorter using the SCS probe (9 min. vs. 17 min.; *p* = 0.001).

In terms of handling, the surgeons preferred the SCS probe in all cases (13/13). This was mainly due to the large size and rigidity of the grid electrode.

The cost of using the grid electrode, a disposable product, was 739 € per patient. The cost per patient for using the SCS probe, a multiple-use product (30 × sterilizable), was 60 €.

### Clinical data

Preoperatively, the overall performance was predominantly well with a median McS of 1 (1–3). Only three patients had severe ataxia preoperatively (median SARA score 1 (0–14)). Except for two patients, all had preoperative pallesthesia impairment in the lower extremity.

There was a worsening of the overall performance according to the McS immediately after surgery (*p* = 0.003). The JOA score worsened significantly after surgery (*p* = 0.007) as well as the pallesthesia of the right upper extremity (*p* = 0.025) and of both lower extremities (right *p* = 0.003; left *p* = 0.002). The ataxic symptoms according to the SARA score did not deteriorate significantly (*p* = 0.07).

Of the 3 patients with worsening SSEPs after myelotomy, 2/3 (67%) had a relevant impairment of neurological and especially DC function preoperatively. These patients did not experience concomitant MEP worsening suggestive of a major vascular event during surgery. Postoperatively, all 3 patients showed a deterioration of DC function and overall performance. In 2 of these 3 patients, both methods identified the midline consistently without deviating from the anatomic midline. In one of these three patients, only the phase reversal method was able to identify the midline even in agreement with the anatomic midline.

Long-term follow-up after a median of 40.5 (range 14–50) months could be obtained in 12 of 13 patients, as one patient died of his progressive tumor disease within the first 12 months after surgery.

**Fig. 4:**
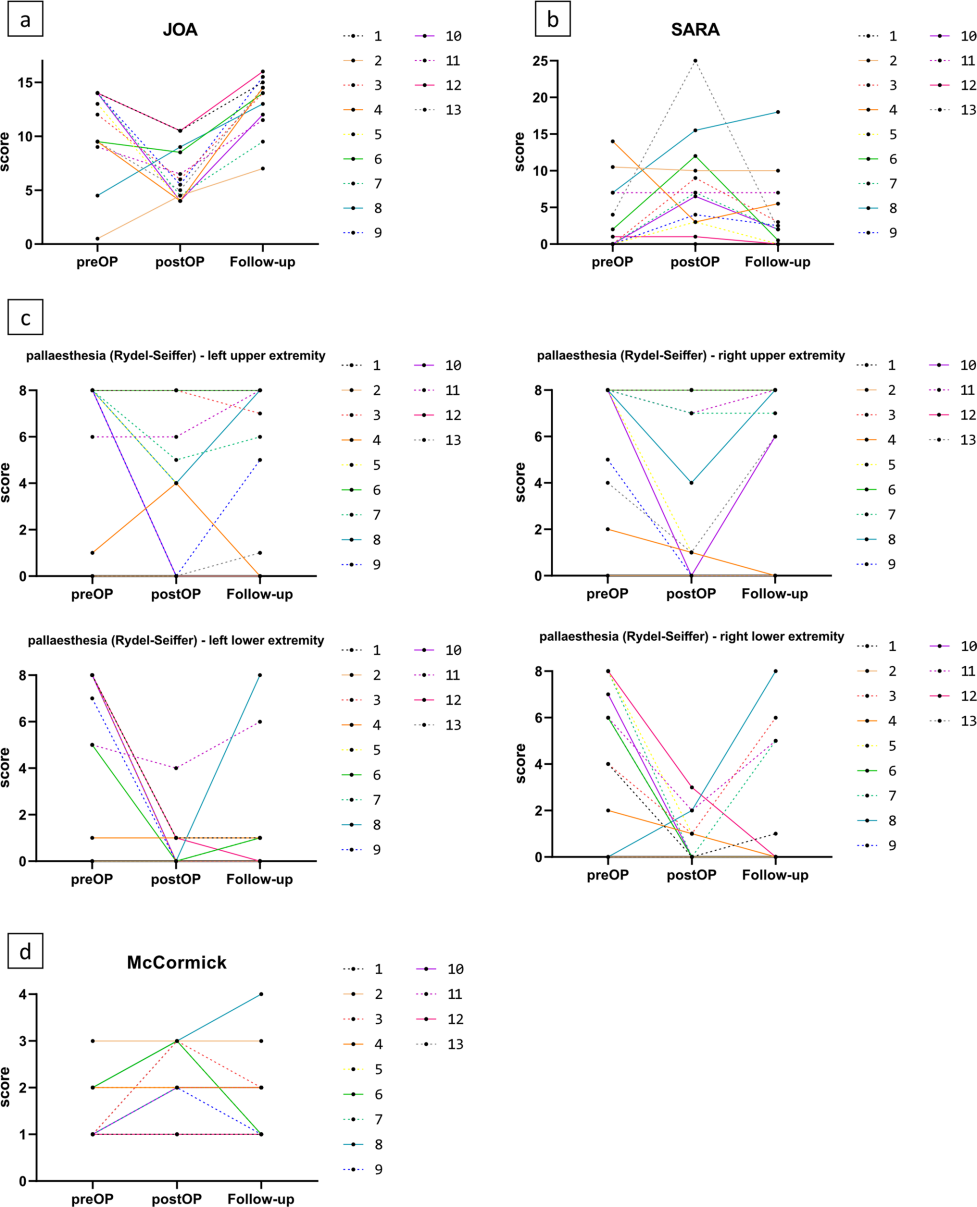
**a** Clinical course according to the JOA score. **b** Clinical course according to the SARA score. **c** Clinical course of pallesthesia of upper and lower extremities. **d** Clinical course according to the McCormick score

The JOA score improved significantly to the postoperative status in 12/12 (100%) patients (*p* < 0.001) and even scored higher values than preoperatively in 10/12 (80%) patients (*p* = 0.025) (Fig. 4A). The SARA score showed an improvement in 5/12 (42%) patients, stable symptoms in 2/12 (17%) patients, and mostly discreet worsening in 5/12 (42%) patients compared to preoperative values without statistical significance (Fig. 4B). With respect to the entire group, pallesthesia of the left lower extremity improved (*p* = 0.016), whereas impairment of the other extremities persisted (Fig. 4C). This observation was independent of tumor extension to the right or left side.

McS remained stable at follow-up compared to the preoperative status in 9/12 (75%), worsened in 2/12 (17%), and improved in 1/12 (8%) patients. The score at long-term follow-up was not significantly different from preoperative status (*p* = 0.08) (Fig. 4D).

According to the clinical course, QoL (SF-36) deteriorated at discharge compared to admission (43.1 vs. 31.1; *p* = 0.0017). Over the long term, however, there was a significant improvement in QoL (31.1 vs. 46.7; *p* = 0.0001), which returned to the preoperative level (43.1 vs. 46.7; *p* = 0.29), indicating a physical and mental health in the average population range (50 ± 10.0).

Both patients with diffuse midline glioma received adjuvant radio chemotherapy but died from their progressive tumor disease. One patient developed a recurrence of his arachnoid cyst but refused to have another surgery. These patients showed no clear clinical deterioration associated with tumor progression. All other patients remained without tumor recurrence.

## Discussion

### Key results

This is the first study to prospectively investigate and compare both methods for dorsal column mapping in imSCT.

Electrophysiological detection of the midline was feasible in 12/13 (92%) patients using either the handheld stimulation device or the grid electrode. In 6 patients, both methods were able to identify the midline simultaneously and consistently. In 3/13 (23.1%) patients, only SCS and, in another 3/13 (23.1%) patients, only spinal SSEP could indicate the midline. Concordance with the anatomical midline was high in these patients. Nevertheless, dorsal column mapping helped to reevaluate and correct the anatomic midline before myelotomy in 1 patient, resulting in stable SSEPs after myelotomy.

The handheld stimulation probe offered significant advantages over the grid electrode in terms of handling and costs.

After postoperative deterioration of the DC function, the long-term course showed a significant recovery of both DC function and QoL.

### Limitations

Due to the rarity of imSCT and the complex comparison of methods, the number of patients in the study is small. Patient recruitment was stopped after a clear preference for the use of the stimulation probe was shown with comparable electrophysiological results of both methods.

Although, the study of Mehta et al. [15] did not find a correlation between tumor histology and DC dysfunction in their series, the heterogeneity of the included tumor entities might have an influence on the surgical strategy. Clinical impairments of the DC function in particular cannot be exclusively attributed to the location of myelotomy. However, there were only two patients with deterioration of SSEPs during tumor resection. Furthermore, thermal injury or indirect injury to the DC from microvascular affection can theoretically mimic direct posterior column injury. In this regard, there were no concurrent MEP losses that could have indicated at least a major vascular problem.

### Interpretation and generalizability

Previous studies have demonstrated the technical feasibility of both methods for DCM in small patient cohorts [15, 23, 28]. After Deletis and Bueno De Camargo [3] developed the technique of DCM via spinal SSEP in 2001, Yanni et al. [28] retrospectively analyzed their experience of the use of this method in 10 patients. They concluded that the technique is safe and effective and leads to satisfying preservation of DC function in their cohort.

In 2012, Simon et al. [23] described the feasibility of the phase reversal method by SCS in one patient, and in 2014, Nair et al. [16] demonstrated the safe and reliable use of a refined technique in 12 patients. Mehta et al. [15] sought to evaluate the clinical impact of the SCS in a retrospective cohort of 11 patients undergoing resection of imSCT with DCM and 80 patients without DCM. They found that DC function was significantly better in the cohort in which DCM was used.

Documentation of unsuccessful application of DCM methods is lacking in the publications cited above. For both DCM methods, preexisting higher-grade impairment of DC function without baseline SSEPs must be noted as a limitation of electrophysiological measurement.

In four patients, no midline could be identified by the use of the stimulation probe. Three of the four tumors were located in the upper cervical cord between C1 and C3.

We assume that in these cases, stimulation cranial to the tumor center was impaired due to the deeper course of the fibers of the gracile and cuneate tracts and the close proximity to the fiber crossing in the medulla oblongata. The correlation analysis between tumor location and measurement failure was not significant. However, the number of patients was small, and the results of the correlation analysis should be reevaluated in studies of larger patient cohorts.

In contrast, in patients with recurrent tumors, we saw a correlation to unsuccessful measurements. Therefore, in recurrent tumors and/or tumors located at the upper cervical spine, close to the craniocervical junction, the direct dorsal column stimulation may not provide reliable measurements. Interestingly, neither the size of the tumor nor the presence or absence of cystic components seemed to play a role for the feasibility of measurements.

In our experience, the main limitation of the grid electrode used for DCM is its size and difficult handling in small and deep surgical approaches, which are commonly performed in the upper cervical spine. However, even in more extended approaches, the difficult handling resulted in a prolongation of the surgical time compared to the stimulation probe. This problem has not been mentioned in previous studies [3, 17]. Possibly, this can be explained by the focus on electrophysiological aspects and the very limited number of cases in these publications. However, this problem could be addressed by the manufacturer using more flexible materials.

In comparison, the stimulation probe was significantly handier, and its easy and flexible use could be advantageous in tumors extending over multiple spinal segments.

Mehta et al. reported an improvement in clinical outcome in 11 consecutive patients who underwent surgery using the grid electrode compared with a historical cohort of patients [15]. In addition to the retrospective study design and the first postoperative examination 3 months after surgery, the lack of a standardized and specific protocol for the study of DC function makes comparisons with our results difficult. Nevertheless, these data and our experience demonstrate the favorable clinical outcome of DC function in patients with imSCT undergoing DCM-guided tumor resection in the mid and long term.

Unlike previous studies, the surgeon had to mark the assumed anatomical midline in all patients in order to allow a comparison to the midline identified by electrophysiology. We found that there was a high concordance between the anatomical and electrophysiological midline. However, in one case, location of the myelotomy was revisited according to the electrophysiological measurements with stable SSEPs after myelotomy. This shows that DCM can be helpful for the confirmation of the anatomical midline as well as for correction of the location of myelotomy in certain cases. Although no complications occurred with the use of either method, one patient required repositioning of the grid electrode because of visible vascular compression at the dorsal surface of the spinal cord. All in all, the use of the stimulation probe will be preferred in our daily routine in the future. However, the disadvantages of the grid electrode could be largely eliminated by improving the materials by the manufacturer. In addition, in cases where there is uncertainty of the anatomical midline and one mapping method does not provide a reliable measurement, the alternative mapping method may be used successfully.

Regarding the clinical course, most patients experienced early postoperative deterioration of posterior column function despite the use of both dorsal column mapping methods. Pallesthesia especially turned out to be at risk for persistent impairment. Other symptoms like ataxia or the epicritical sensitivity showed significant improvement over time. This clinical improvement argues for sparing of the gracile and cuneate fasciculi. The concomitant positive evolution of SF-36 scores after surgery may reflect the importance of DC function for patients’ QoL.

## Conclusion

Electrophysiological detection of the midline for myelotomy can help the surgeon to confirm or revisit the anatomical identification in patients with stable preoperative SSEPs. Upper cervical tumor location and small surgical approaches appear to prevent successful midline detection with the grid electrode. Recurrent tumors seem to complicate successful midline detection with the simulation probe. While both mapping methods are equivalent in terms of midline detection, the phase reversal method revealed advantages of dorsal column mapping in terms of handling and costs.

After early postoperative worsening of symptoms, both long-term clinical outcomes and QoL were favorable.

### Supplementary Information

Below is the link to the electronic supplementary material.

Supplementary file1 (DOCX 27 KB)

## References

[1] Bickerton RC, Barr GS (1987). The origin of the tuning fork. J R Soc Med.

[2] Cristante L, Herrmann HD (1994). Surgical management of intramedullary spinal cord tumors functional outcome and sources of morbidity. Neurosurgery.

[3] Deletis V, Bueno De Camargo A (2001). Interventional neurophysiological mapping during spinal cord procedures. Stereotact Funct Neurosurg.

[4] Epstein FJ, Farmer JP, Freed D (1993). Adult intramedullary spinal cord ependymomas: the result of surgery in 38 patients. J Neurosurg.

[5] Forster MT, Marquardt G, Seifert V, Szelenyi A 2012 Spinal cord tumor surgery importance of continuous intraoperative neurophysiological monitoring after tumor resection Spine Phila Pa 1976 37 E1001–1008 10.1097/BRS.0b013e31824c76a8 10.1097/BRS.0b013e31824c76a8 22322374

[6] Fukui M Chiba K Kawakami M Kikuchi S Konno S Miyamoto M Seichi A Shimamura T Shirado O Taguchi T Takahashi K Takeshita K Tani T Toyama Y Wada E Yonenobu K Tanaka T Hirota Y 2007 Subcommittee on Low Back P, Cervical Myelopathy Evaluation of the Clinical Outcome Committee of the Japanese Orthopaedic A An outcome measure for patients with cervical myelopathy: Japanese Orthopaedic Association Cervical Myelopathy Evaluation Questionnaire (JOACMEQ) Part 1 Journal of orthopaedic science official journal of the Japanese Orthopaedic Association 12 227 240 10.1007/s00776-007-1118-1 10.1007/s00776-007-1118-1 PMC2778723 17530374

[7] Garrido E, Stein BM (1977). Microsurgical removal of intramedullary spinal cord tumors. Surg Neurol.

[8] Grimm S, Chamberlain MC (2009). Adult primary spinal cord tumors. Expert Rev Neurother.

[9] Hoshimaru M, Koyama T, Hashimoto N, Kikuchi H (1999). Results of microsurgical treatment for intramedullary spinal cord ependymomas: analysis of 36 cases. Neurosurgery.

[10] Juthani RG, Bilsky MH, Vogelbaum MA (2015). Current management and treatment modalities for intramedullary spinal cord tumors. Curr Treat Options Oncol.

[11] Klekamp J (2013). Treatment of intramedullary tumors: analysis of surgical morbidity and long-term results. J Neurosurg Spine.

[12] Kothbauer KF, Deletis V, Epstein FJ 1998 Motor-evoked potential monitoring for intramedullary spinal cord tumor surgery correlation of clinical and neurophysiological data in a series of 100 consecutive procedures Neurosurg Focus 4 e1 10.3171/foc.1998.4.5.4 10.3171/foc.1998.4.5.4 17154450

[13] Manzano G, Green BA, Vanni S, Levi AD (2008). Contemporary management of adult intramedullary spinal tumors-pathology and neurological outcomes related to surgical resection. Spinal cord.

[14] McCormick PC, Torres R, Post KD, Stein BM (1990). Intramedullary ependymoma of the spinal cord. J Neurosurg.

[15] Mehta AI, Mohrhaus CA, Husain AM, Karikari IO, Hughes B, Hodges T, Gottfried O, Bagley CA (2012). Dorsal column mapping for intramedullary spinal cord tumor resection decreases dorsal column dysfunction. J Spinal Disord Tech.

[16] Nair D, Kumaraswamy VM, Braver D, Kilbride RD, Borges LF, Simon MV (2014). Dorsal column mapping via phase reversal method the refined technique and clinical applications. Neurosurgery.

[17] Quinones-Hinojosa A, Gulati M, Lyon R, Gupta N, Yingling C (2002). Spinal cord mapping as an adjunct for resection of intramedullary tumors surgical technique with case illustrations. Neurosurgery.

[18] Sala F, Krzan MJ, Deletis V (2002). Intraoperative neurophysiological monitoring in pediatric neurosurgery: why, when, how?. Childs Nerv Syst.

[19] Samartzis D, Gillis CC, Shih P, O'Toole JE, Fessler RG (2016). Intramedullary spinal cord tumors: part II-management options and outcomes. Global spine journal.

[20] Sandalcioglu IE, Gasser T, Asgari S, Lazorisak A, Engelhorn T, Egelhof T, Stolke D, Wiedemayer H (2005). Functional outcome after surgical treatment of intramedullary spinal cord tumors: experience with 78 patients. Spinal cord.

[21] Schmitz-Hubsch T, du Montcel ST, Baliko L, Berciano J, Boesch S, Depondt C, Giunti P, Globas C, Infante J, Kang JS, Kremer B, Mariotti C, Melegh B, Pandolfo M, Rakowicz M, Ribai P, Rola R, Schols L, Szymanski S, van de Warrenburg BP, Durr A, Klockgether T, Fancellu R (2006). Scale for the assessment and rating of ataxia: development of a new clinical scale. Neurology.

[22] Shrivastava RK, Epstein FJ, Perin NI, Post KD, Jallo GI (2005). Intramedullary spinal cord tumors in patients older than 50 years of age: management and outcome analysis. J Neurosurg Spine.

[23] Simon MV, Chiappa KH, Borges LF (2012). Phase reversal of somatosensory evoked potentials triggered by gracilis tract stimulation: case report of a new technique for neurophysiologic dorsal column mapping. Neurosurgery.

[24] Skrap B, Tramontano V, Faccioli F, Meglio M, Pinna G, Sala F 2021 Surgery for intramedullary spinal cord ependymomas in the neuromonitoring era results from a consecutive series of 100 patients J Neurosurg Spine 1–11 10.3171/2021.7.SPINE21148 10.3171/2021.7.SPINE21148 34891138

[25] Stein BM (1979). Surgery of intramedullary spinal cord tumors. Clin Neurosurg.

[26] Ware JE, Sherbourne CD (1992). The MOS 36-item short-form health survey (SF-36). I. Conceptual framework and item selection. Med Care.

[27] William D. Willis, CHAPTER 348 - Retrograde signaling in the nervous system: dorsal root reflexes, Editor(s): Ralph A. Bradshaw, Edward A. Dennis, Handbook of Cell Signaling, Academic Press, 2003 Pages 607–614 ISBN 9780121245467 10.1016/B978-012124546-7/50712-9

[28] Yanni DS, Ulkatan S, Deletis V, Barrenechea IJ, Sen C, Perin NI (2010). Utility of neurophysiological monitoring using dorsal column mapping in intramedullary spinal cord surgery. J Neurosurg Spine.

