# Supplementary material for: Dorsal column mapping in resection of intramedullary spinal cord tumors: a prospective comparison of two methods and neurological follow-up
Source: Acta Neurochir (Wien). 2023 Mar 17;165(11):3493–504. doi: 10.1007/s00701-023-05554-1 (PMC10624746; doi:10.1007/s00701-023-05554-1)
Supplement: Supplementary file 1 — Supplementary file1 (DOCX 27 KB) [file 701_2023_5554_MOESM1_ESM.docx]

**DCM- intraoperative protocol**

Patient label

#1 **Grid electrode median nerve**

Diagnosis:

Surgery:

Date:

Surgeon:

Start _______ end ______ of stimulation

Impedance: ....................................Ohm

Stimulation intensity: ......................................mA

Stimulation frequency: ....................................... Hz

Stimulation duration: ........................................sec

Filter settings: .........................................

**DCM- intraoperative protocol**

**#1 Stimulation median nerve right (r)/left (l)**

| Location of grid | Electrodes |
| --- | --- |
| Cranial  Max. amplitudes | ☐ ☐ ☐ ☐ ☐ ☐ ☐ ☐ right  1 2 3 4 5 6 7 8  ☐ ☐ ☐ ☐ ☐ ☐ ☐ ☐ left  1 2 3 4 5 6 7 8 |
| Medial  Max. amplitudes | ☐ ☐ ☐ ☐ ☐ ☐ ☐ ☐ right  1 2 3 4 5 6 7 8  ☐ ☐ ☐ ☐ ☐ ☐ ☐ ☐ left  1 2 3 4 5 6 7 8 |
| Caudal  Max. amplitudes | ☐ ☐ ☐ ☐ ☐ ☐ ☐ ☐ right  1 2 3 4 5 6 7 8  ☐ ☐ ☐ ☐ ☐ ☐ ☐ ☐ left  1 2 3 4 5 6 7 8 |
| Stimulation possible | Y ☐ N ☐ |
| Handling preference of the surgeon | ☐ |
| Photo documentation | Y ☐ N ☐ |

**DCM- intraoperative protocol**

**#1 Stimulation tibial nerve right (r)/left (l)**

Start _______ end ______ of stimulation

Impedance: ....................................Ohm

Stimulation intensity: ......................................mA

Stimulation frequency: ....................................... Hz

Stimulation duration: ........................................sec

Filter settings: .........................................

| Location of grid in relation to the tumor | Electrodes |
| --- | --- |
| Cranial  Max. amplitudes | ☐ ☐ ☐ ☐ ☐ ☐ ☐ ☐ right  1 2 3 4 5 6 7 8  ☐ ☐ ☐ ☐ ☐ ☐ ☐ ☐ left  1 2 3 4 5 6 7 8 |
| Medial  Max. amplitudes | ☐ ☐ ☐ ☐ ☐ ☐ ☐ ☐ right  1 2 3 4 5 6 7 8  ☐ ☐ ☐ ☐ ☐ ☐ ☐ ☐ left  1 2 3 4 5 6 7 8 |
| Caudal  Max. amplitudes | ☐ ☐ ☐ ☐ ☐ ☐ ☐ ☐ right  1 2 3 4 5 6 7 8  ☐ ☐ ☐ ☐ ☐ ☐ ☐ ☐ left  1 2 3 4 5 6 7 8 |
| Stimulation possible | Y ☐ N ☐ |

Comments:

|  |
| --- |

**DCM- intraoperative protocol**

#2 **Stimulation probe**

Start _______ end ______ of stimulation

| Location of grid in relation to the tumor | C3‘/C4‘ phase reversal with respect to normal N20/P40 polarity | C4‘/C3‘ phase reversal with respect to normal N20/P40 polarity |
| --- | --- | --- |
| Cranial |  |  |
| Medial |  |  |
| Caudal |  |  |
| Spinal SSEPs right  Spinal SSEPs left | Y ☐ N ☐  Y ☐ N ☐ |  |
| Handling preference of the surgeon | ☐ |  |
| Photo documentation | Y ☐ N ☐ |  |

Comments:

|  |
| --- |

Fig. 4: Intraoperative protocol for the documentation of dorsal column mapping.
